# Which Is the Optimum Antigen Concentration for the Venereal Disease Research Laboratory Test of Cerebrospinal Fluid for Neurosyphilis Diagnosis: 10 or 17 μL?

**DOI:** 10.3389/fmed.2022.877186

**Published:** 2022-04-28

**Authors:** Yao Xiao, Wei Li, Qiu-Ling Li, Qiu-Yan Xu, Yang Yang, Tian-Ci Yang, Li-Li Liu, Wei-Ming Gu

**Affiliations:** ^1^Center of Clinical Laboratory, Zhongshan Hospital of Xiamen University, School of Medicine, Xiamen University, Xiamen, China; ^2^Department of Hospital Infection Management, Zhongshan Hospital of Xiamen University, School of Medicine, Xiamen University, Xiamen, China; ^3^Institute of Infectious Disease, School of Medicine, Xiamen University, Xiamen, China; ^4^Shanghai Skin Disease Hospital, Tongji University School of Medicine, Shanghai, China

**Keywords:** CSF, VDRL, antigen concentration, neurosyphilis, diagnosis

## Abstract

The manufacturer's instructions for the venereal disease research laboratory (VDRL) antigen test for diagnosing neurosyphilis describe testing of serum samples and do not include procedures for cerebrospinal fluid (CSF) testing. This study compared the CSF-VDRL test with 10 μL of antigen (CSF-VDRL-10) according to the American Public Health Association to the CSF-VDRL test with 17 μL of antigen (CSF-VDRL-17) according to the VDRL serum procedure. A total of 121 neurosyphilis patients and 86 syphilis/non-neurosyphilis patients were included. The sensitivities of the CSF-VDRL-10 and CSF-VDRL-17 tests were comparable for neurosyphilis diagnosis. The positive rate of the CSF-VDRL-17 test was higher than that of the CSF-VDRL-10 test. In all, 78.3% of the quantitative CSF-VDRL-17 results were consistent with those of the CSF-VDRL-10 test, 18.4% exhibited one-titer higher results than those of the CSF-VDRL-10 test, and 3.4% had positive CSF-VDRL-17 results but negative CSF-VDRL-10 results. The CSF-VDRL test with 17 μL of antigen was more sensitive, and it is worth performing longitudinal studies to understand its practical implications.

## Introduction

The cerebrospinal fluid venereal disease research laboratory (CSF-VDRL) test remains the current standard for the laboratory diagnosis of neurosyphilis (1–3). However, according to the manufacturer's instructions, a VDRL antigen concentration of 17 μL is recommended for serum sample testing (4). With modification, this procedure can be used for testing CSF samples. The VDRL test procedures for CSF samples, in which 10 μL of VDRL antigen is used, were modified from those for serum samples by the American Public Health Association (5). The present study aimed to further compare the operating procedures of the VDRL method for CSF testing at antigen concentrations of 17 and 10 μL.

## Methods

Between August 2017 and January 2021, 207 patients at Zhongshan Hospital, School of Medicine, Xiamen University, were suspected of having neurosyphilis and received non-treponemal tests and treponemal tests of both serum and CSF samples. This study was approved by the Ethics Committee of the School of Medicine, Xiamen University, and it was conducted in compliance with national legislation of China and the Declaration of Helsinki guidelines. Written informed consent was obtained from all the study participants.

The diagnostic criteria for neurosyphilis complied with the guidelines of the Centers for Disease Control in the United States and Europe (6, 7). Briefly, neurosyphilis was defined as syphilis at any stage with a positive *Treponema pallidum* particle agglutination (TPPA) assay result based on a CSF sample and a combination of the following findings: (1) reactive VDRL in a CSF sample, (2) clinical symptoms or signs consistent with neurosyphilis without other known causes for these clinical abnormalities, and (3) elevated CSF protein concentration (>500 mg/L) or leukocyte count (>5 cells/μL) in the absence of other known causes of these abnormalities. Verified neurosyphilis was defined as meeting conditions (1) and (2); likely neurosyphilis was defined as meeting conditions (2) and (3); and possible neurosyphilis was defined as meeting condition (3). The above three classifications were all treated as neurosyphilis.

Serological tests for syphilis were performed on all participants using a toluidine red unheated serum (TRUST) test (Shanghai Rongsheng Biotech Co., Ltd. Shanghai, China) and TPPA tests (Fujirebio, Tokyo, Japan) according to the manufacturer's instructions and as previously reported (8). The VDRL test (Becton, Dickinson Co., Maryland, USA) for CSF samples was performed in a manner similar to that of the test for serum samples (5): initially, a qualitative test was performed by placing 50 μL of the specimen into one 16 mm ring of a ceramic-ringed slide and then adding one drop of antigen. Next, the slide was placed on a mechanical rotator and rotated at 180 rpm. Immediately after rotation, the slide was read with a light microscope. Unlike the serum samples, the CSF samples were not heated before the test; additionally, in contrast to the serum test procedures, in the CSF test procedures, the antigen drop volume was 10 μL, and the slide was rotated for 8 min. The quantitative test was performed in a manner comparable to the qualitative test, except that serial 2-fold dilutions of the samples were prepared in 0.9% saline. Quantitative results were reported as the highest dilution giving a reactive result. The CSF-VDRL test with 10 μL of antigen is routine in clinical practice and is referred to as the CSF-VDRL-10 test in this study. The routine CSF-VDRL test was modified according to the serum procedure with 17 μL of antigen and is referred to as the CSF-VDRL-17 test.

The diagnostic performance of the CSF-VDRL-10 and CSF-VDRL-17 tests was determined and compared to the results of diagnostic criteria for neurosyphilis, according to the Yerushalmy model. McNemar's χ^2^ test was used to test the difference between paired proportions. All statistical analyses were performed using SPSS 25.0 for Windows (SPSS Inc., Chicago, IL, USA). A two-sided *P* value of <0.05 was considered statistically significant.

## Results

A total of 207 participants were included in the study, namely, 121 neurosyphilis patients and 86 syphilis/non-neurosyphilis patients. The clinical characteristics of the study participants are shown in Table 1. The sensitivities of the CSF-VDRL-10 and CSF-VDRL-17 tests for neurosyphilis diagnosis were compared for the neurosyphilis patients and were 71.1 (86/121) and 74.4% (90/121), respectively. There was no significant difference in the sensitivity between the two tests (*P* = 0.125) (Table 2). The specificities of the CSF-VDRL-10 and CSF-VDRL-17 tests for a neurosyphilis diagnosis were compared for the syphilis/non-neurosyphilis patients, with values of 100.0% (86/86) and 96.5% (83/86), respectively. McNemar's χ^2^ test between the two tests in terms of specificity could not be performed because the number of rows and columns of the crosstab was <2 (Table 2). The positive rate of the qualitative CSF-VDRL-17 test was 44.9% (93/207), which was higher than that of the qualitative CSF-VDRL-10 test (41.5% [86/207]) (*P* = 0.016). There were seven patients who had negative CSF-VDRL-10 results but positive CSF-VDRL-17 results. The agreement rate between the two tests was 96.6% (200/207).

**Table 1:** Clinical characteristics of the study participants.

	**Neurosyphilis (*n* = 121)**	**Syphilis/Non-neurosyphilis (*n* = 86)**	**Whole cohort (*n* = 207)**
Sex ratio (male: female)	88:33	38:48	126:81
Age, median years (range)	51 (25–79)	42 (21–84)	47 (21–84)
Positive CSF-VDRL-10, *n* (%)	86 (71.1)	0 (0.0)	86 (41.5)
Positive CSF-VDRL-17, *n* (%)	90 (74.4)	3 (3.5)	93 (44.9)

*CSF, cerebrospinal fluid; VDRL, venereal disease research laboratory*.

**Table 2:** Comparison of the sensitivities and specificities between the CSF-VDRL-10 and CSF-VDRL-17 tests for neurosyphilis diagnosis.

	**Sensitivity comparison in neurosyphilis patients (*****n*** **=** **121)**			**Specificity comparison in syphilis/non-neurosyphilis patients (*****n*** **=** **86)**		
**CSF-VDRL-17**	**CSF-VDRL-10**		***P***	**CSF-VDRL-10**		***P***
	**Positive**	**Negative**		**Positive**	**Negative**	
Positive	86	4	0.125	0	3	n.a.
Negative	0	31		0	83	

*n.a., not applicable. McNemar's χ^2^ test between the two tests in terms of specificity could not be performed because the number of rows and columns of the crosstab was <2*.

The probability distribution of the quantitative CSF-VDRL-17 results was significantly different from that of the quantitative CSF-VDRL-10 results (*P* < 0.001). Compared with that of the quantitative CSF-VDRL-10 test, 78.3% (162/207) of the quantitative CSF-VDRL-17 results were in complete agreement, including 114 patients having negative results and 48 patients having the same titers (Table 3). For the other 18.4% (38/207) of the patients who had positive results in both tests, their quantitative CSF-VDRL-17 results were one-titer higher than those for the CSF-VDRL-10 test. For the remaining 3.4% (7/207) of the patients who had positive CSF-VDRL-17 results but negative CSF-VDRL-10 results, the quantitative CSF-VDRL-17 results were all 1:1. According to the diagnostic criteria, four of these patients were diagnosed with neurosyphilis according to their neurological symptoms and elevated CSF leukocyte count, and three of these patients were not diagnosed with neurosyphilis due to a lack of neurological symptoms.

**Table 3:** The agreement between the qualitative and quantitative CSF-VDRL-10 and CSF-VDRL-17 tests.

**CSF-VDRL-17**	**CSF-VDRL-10**										
	**Negative**	**1:1**	**1:2**	**1:4**	**1:8**	**1:16**	**1:32**	**1:64**	**1:128**	***P*** **for *χ^2^***	**Agreement**
**Whole cohort (*****n*** **=** **207)**											
Negative	114	0	0	0	0	0	0	0	0	<0.001	78.3%
1:1	7	14	0	0	0	0	0	0	0		
1:2	0	12	11	0	0	0	0	0	0		
1:4	0	0	12	6	0	0	0	0	0		
1:8	0	0	0	7	13	0	0	0	0		
1:16	0	0	0	0	4	2	0	0	0		
1:32	0	0	0	0	0	3	1	0	0		
1:64	0	0	0	0	0	0	0	0	0		
1:128	0	0	0	0	0	0	0	0	1		

*CSF, cerebrospinal fluid; VDRL, venereal disease research laboratory*.

## Discussion

The VDRL assay is a non-treponemal test that detects antibodies (i.e., reagin) to lipoidal antigens (i.e., cardiolipin) released from the treponemal cell surface and damaged host cells through a flocculation reaction (9). To date, the CSF-VDRL test remains the only recommended test for the laboratory diagnosis of neurosyphilis (1). However, the manufacturer's instructions recommend the use of the reagent for serum sample testing (4). Thus, this study evaluated the CSF-VDRL test with different concentrations of antigen to diagnose neurosyphilis.

Our findings showed that the sensitivity of the CSF-VDRL-17 test for neurosyphilis diagnosis was comparable to that of the CSF-VDRL-10 test. Moreover, the positive rate of the CSF-VDRL-17 test was significantly higher than that of the routine CSF-VDRL-10 test. Four patients with positive CSF-VDRL-17 but negative CSF-VDRL-10 results were diagnosed with neurosyphilis. A larger concentration of antigen may promote antigen-antibody lattice formation, indicating a better sensitivity of the CSF-VDRL-17 test for patients with very low concentrations of antibodies.

Moreover, three patients with discordant results were not diagnosed with neurosyphilis, because a reactive CSF-VDRL-10 test was the only diagnostic criterion for neurosyphilis for syphilis patients without neurological symptoms or other CSF abnormalities. The discordant results may be due to that the probability of false positives increased as the concentration of antigen in the CSF-VDRL-17 test increased. Antilipoidal antibodies are produced not only as a consequence of syphilis and other treponemal diseases but also in response to non-treponemal diseases of an acute and chronic nature in which tissue damage occurs (10). Although the incidence of biological false-positive VDRL tests is rare in spinal fluid samples (11), some studies have reported false-positive CSF VDRL results in the setting of central nervous system malignancy or HIV infection (12–14). However, for these syphilis patients, negative CSF-VDRL-10 results but positive CSF-VDRL-17 results may be indications for a very early phase of asymptomatic neurosyphilis and contribute to early diagnosis. A continuous follow-up visit was needed to verify it.

In conclusion, our study demonstrated that the CSF-VDRL test with 10 or 17 μL of antigen had comparable diagnostic performance for neurosyphilis diagnosis. The CSF-VDRL test with 17 μL of antigen was more sensitive than that with 10 μL, and it is worth performing longitudinal studies to understand its practical implications.

## Data Availability Statement

The raw data supporting the conclusions of this article will be made available by the authors, without undue reservation.

## Ethics Statement

The studies involving human participants were reviewed and approved by Ethics Committee of School of Medicine, Xiamen University. The patients/participants provided their written informed consent to participate in this study.

## Author Contributions

YX and WL wrote the main manuscript text. Q-LL and Q-YX contributed to sample collection and laboratory tests. YY and T-CY performed the statistical analysis. L-LL and W-MG conceived the study and revised the manuscript. All authors contributed to the article and approved the submitted version.

## Funding

This work was supported by the National Natural Science Foundation of China (Grant Nos. 82003512, 81973104, 81772260, and 81971147), the Key Projects for Province Science and Technology Program of Fujian Province, China (Grant No. 2018D0014), and the Natural Science Foundation of Fujian Province, China (Grant No. 2021J02055).

## Conflict of Interest

The authors declare that the research was conducted in the absence of any commercial or financial relationships that could be construed as a potential conflict of interest.

## Publisher's Note

All claims expressed in this article are solely those of the authors and do not necessarily represent those of their affiliated organizations, or those of the publisher, the editors and the reviewers. Any product that may be evaluated in this article, or claim that may be made by its manufacturer, is not guaranteed or endorsed by the publisher.
